# Does working in an extremely cold environment affects lung function?: 10 years follow-up

**DOI:** 10.1007/s00420-023-01988-3

**Published:** 2023-06-03

**Authors:** Marcial Velasco Garrido, Nadine Rentel, Robert Herold, Volker Harth, Alexandra M. Preisser

**Affiliations:** Institute for Occupational and Maritime Medicine (ZfAM), University Medical Center Hamburg-Eppendorf, Seewartenstr. 10, 20459 Hamburg, Germany

**Keywords:** Cold exposure, Lung function, Occupational exposure, Pulmonary disease, Long-term

## Abstract

**Objective:**

The aim of this study is to investigate whether there is an association between brief but repeated exposures to extremely cold temperatures over many years and pulmonary function.

**Methods:**

We performed a retrospective analysis of the data collected over 10 years in the context of the extended medical examinations of storeworkers exposed to extremely cold temperatures. We considered forced vital capacity (FVC), forced expiratory volume in one second (FEV_1_), Tiffeneau-Pinelli index (FEV_1_/FVC), CO diffusion capacity (D_L,CO_) and Krogh-factor (CO diffusion capacity relative to recorded alveolar volume, D_L,CO_/VA) reported as %-predicted. We analysed trends in outcome parameters with linear mixed models.

**Results:**

46 male workers participated in at least two extended medical examinations between 2007 and 2017. Overall 398 measure points were available. All lung function parameters had values above the lower limit of normality at the first examination. In the multivariate model including smoking status and monthly intensity of cold exposure (≤ 16 h/month vs. > 16 h/month) FEV1%-predicted and FVC %-predicted had a statistically significant positive slope (FEV1, 0.32% 95% CI 0.16% to 0.49% *p* < 0.001; FVC 0.43% 95% CI 0.28% to 0.57% *p* < 0.001). The other lung function parameters (FEV1/FVC %-predicted, DL,CO %-predicted, DL,CO/VA %-predicted) showed no statistically significant change over time.

**Conclusions:**

Long term intermittent occupational exposure to extreme cold temperatures (-55 °C) does not appear to cause irreversible deleterious changes in lung function in healthy workers, thus the development of obstructive or restrictive lung diseases is not expected.

## Introduction

The human physiological reaction to cold temperatures to keep homeostatic body temperature is well known and includes peripheral vasoconstriction with cooling of dermal temperature, shivering, and an increase in respiratory rate (Granberg 1991). Short-term response to exposure against cold air comprises rhinorrhoea, nasal obstruction, cough and bronchoconstriction (Koskela 2007). In particular, individuals with bronchial asthma report exacerbation of symptoms when exposed to cold weather (Hyrkäs-Palmu et al. 2018). There is evidence that the number of hospitalisations due to asthma and chronic obstructive pulmonary disease increase in very cold days (Chen et al. 2022; Liu et al. 2021). Some studies showed a reduction of the forced expiratory capacity in one second (FEV_1_) in spirometry after cold air exposure in both healthy subjects and individuals with known asthma or COPD (Koskela and Tukiainen 1995; Koskela et al. 1996). However, it is controversial whether breathing cold air only causes respiratory symptoms (Koskela 2007) or whether it may play a role in inducing respiratory diseases by triggering inflammation and airway remodelling in healthy subjects, as has been observed in winter sport athletes (Sue-Chu 2012).

Occupational exposure to low temperatures occurs either while working outdoors (e.g. fishery, forestry, construction) or while working in technically refrigerated rooms (e.g. food processing, storage, transportation) (Groos and Thielmann 2020). Occupational exposure over longer periods to cold temperatures has been shown to be associated with a higher incidence of respiratory symptoms (wheeze and cough) in previously healthy workers (Stjernbrandt et al. 2022). According to DIN 33403–5 ambient temperatures under − 30 °C are considered extremely cold or ultra-cold. Particularly in the food industry, cold- and deep-freeze-storage is required to ensure the preservation and quality of products. Depending on the kind of processing and storage methods, work temperature might reach up to − 60 °C in the food industry (Piedrahita et al. 2008). This implies that several thousand workers worldwide are repeatedly exposed to freeze temperatures in their jobs. Cold storage workers exposed to temperatures between − 20 °C and − 30 °C reported respiratory symptoms (wheeze, shortness of breath, cough, and increased mucus production) significantly more frequently than unexposed workers (Ghani et al. 2020). However, little is known about the effects of occupational indoor cold exposure on spirometry lung function parameters. To our knowledge, only Jammes et al. (2002) demonstrated a decrease in FEV_1_ and an increase in airway resistance in a 12 month follow-up of cold storage workers exposed to a temperature of + 3 °C during 25% of their working hours each day. However, there are no studies addressing lung function (including diffusion capacity) in association with indoor occupational exposure to temperatures below 0 °C over more than one year.

The aim of the present study is to investigate whether there is an association between brief but repeated exposures to extremely cold temperatures over many years and pulmonary function.

## Methods

### Study population

In the year 2007 a factory producing enzymes for the food industry put into operation store-rooms working at a temperature of -55 °C in Northern Germany. Because of concerns regarding potential detrimental effects to health of the regular exposure of workers to such temperatures, the regional supervisory health authority allowed the operation of the installations only under the condition of conducting extensive medical surveillance examinations every six months among the storekeepers being exposed to these extremely low temperatures. The storekeepers are logistic workers who enter the refrigerators several times per day to pick-up the products and prepare them for delivery to food manufacturers. The length of stay in the extreme cold rooms varies between 15 and 30 min per stay depending on the amount of products to be commissioned. The workers wear protective clothes adequate for extremely low temperatures (i.e. polar clothing) but no specific respiratory protective equipment.

### Study design

We performed a retrospective analysis of the data collected between the years 2007 and 2017 in our Institute for Occupational and Maritime Medicine (ZfAM, Hamburg, Germany) in the context of the extended medical examinations ordered by the regional supervisory health authority for the storeworkers exposed to extremely cold temperatures.

To be included in the study, the workers had to be free of known respiratory disease at the beginning of the follow-up (2007) and had to have attended at least three medical examinations in the ten-year period.

### Medical examinations

The extended medical examination was conducted every 6 months at our Institute. At each visit, workers signed-up the form consenting to the use of their medical data for scientific evaluation.

In each visit, workers were asked about the average time of exposure to the extremely cold temperatures in the past week and in the past month. They were also asked about the incidence of cold-related complaints since the last visit and about their smoking status (current smoker, former smoker, never smoker). The answers were recorded in a form.

Besides the medical interview, the visit consisted of a medical general examination including measurement of stature (cm), and pulmonary function tests (spirometry and a measurement of diffusion capacity for carbon monoxide (CO)). At the time of the examination the last occupational exposure to extremely cold temperatures in the store dated back at least 12 h.

### Pulmonary function tests

Spirometry was carried out with a pneumotachograph (*MasterScreen CareFusion Germany 234 GmbH*, Höchberg, Germany, in its consecutive versions) according to the quality criteria of the European Respiratory Society (ERS) (Miller et al. 2005), the American Thoracic Society (ATS) (Pellegrino et al. 2005) and the German guideline for standardization of spirometry (Criée et al. 2015), which require three artefact-free spirometry breathing manoeuvres. The best result of two reproducible manoeuvres was selected. All volumes were measured in litres.

Diffusion capacity for carbon monoxide (D_L,CO_) was measured in mmol/min/kPa by the single breath (SB) method with *MasterScreen Diffusion* (*CareFusion Germany 234 GmbH*, Höchberg, Germany, in its consecutive versions) according to the recommendations of MacIntyre et al. (2005) and Graham et al. (2017). D_L,CO_ values were used only when the inspired volume in the SB-manoeuvre (V_in_) achieved at least 85% of the vital capacity. We performed two manoeuvres. If both were acceptable, the mean of both measurements was taken, otherwise, the best one was used. Since no haemoglobin value (Hb) was available, we standardized all DL_CO_ measurements to a normed Hb value of 14.6 g/dl (which is the default setting of the software) according to the formula D_L,CO_–c = D_L,CO_*(10.22 + Hb)/(1.7*Hb) (Mottram et al. 1999).

### Outcome parameters

For the present study we considered forced vital capacity (FVC), forced expiratory volume in one second (FEV_1_), Tiffeneau-Pinelli index (FEV_1_/FVC), CO diffusion capacity (D_L,CO_) and Krogh-factor (CO diffusion capacity relative to recorded alveolar volume, D_L,CO_ /VA) reported as %-predicted. Predicted values for spirometric parameters were calculated according to the reference equations of the Global Lung Initiative (GLI) (Quanjer et al. 2012). Predicted D_L,CO_ and D_L,CO_/VA were calculated according to the equations of Cotes et al. (1993).

### Statistical analyses

Descriptive statistics are reported as means with standard deviation (SD) for continuous variables, and as frequencies and percentages for categorical variables.

To account for the longitudinal character of the data with repeated measurements over the follow-up period with intraclass correlation at the level of the individual workers, we analysed trends in outcome parameters (%-predicted FCV, %-predicted FEV_1_, %-predicted FEV_1_/FVC, %-predicted D_L,CO_, %-predicted D_L,CO_/VA) with linear mixed models. To capture the individual development over time, the measurements were numbered by the order regardless of the date when they were first performed. Date of the examination 1 was constituted by all first measurements, date of examination 2 by all second ones, and so on up to date of examination 20 (i.e. constituted by the last available measurement of the workers who participated in all examinations during the 10-year period). The measurements are nested within individuals, who represent the random effects of the mixed model. Model fitting was performed stepwise. Basic models included the outcome parameters as a function of time in random intercept, fixed slope models. In the next step we added smoking status (“never” / “former” / “current”) or monthly exposure to extreme cold (“ ≤ 16 h/month” / “ > 16 h/month”) respectively with random intercept and fixed slope. The 16-h cut-off for extreme cold exposure was chosen because during the follow-up period, the health authority revoked the obligation for special extended health surveillance for workers with monthly exposures of 16 h or less, so these workers no longer showed up. Finally, the outcome parameters were modelled as a function of all variables (time, monthly exposure, and smoking status) in a random intercept, fixed slope model. We performed sensitivity analysis with exposure time added as a continuous variable and restricted to the first 7 examinations. We explored other assumptions (fixed intercept/random slope or random intercept/random slope) in the models as recommended (Field 2013). We report models with random intercept and fixed slope. Slopes are presented as change in %-predicted with 95% confidence intervals (CI).

We calculated two-tailed p values. The statistical significance level was set at p < 0.05.

All computations were carried out with IBM® SPSS® Statistics (IBM Corp. Released 2019. IBM SPSS Statistics for Windows, Version 26.0. Armonk, NY: IBM Corp).

### Ethics approval

According to the Ethics Committee of the Hamburg Medical Association no additional approval was required because of the retrospective character of the study with in-house routinely collected data.

## Results

A total of 46 male workers participated in at least three extended medical examinations between 2007 and 2017. The majority of them (58.7%) were exposed to extreme cold temperatures for more than 16 h per month. The number of workers with the respective number of examinations performed is listed in Table 1. Overall 398 measure points where available, 71.4% of the measure points were produced between examination no. 1 and no. 7. The majority of measurements (70.6%) had been done among workers with monthly exposures of more than 16 h. Mean number of examinations per worker was 8.6; 6 workers had 20 examinations (i.e. follow-up of 10 years). Median follow-up was 3.5 year. Mean age at the first examination was 35.09 (SD 9.34) years. Mean age at the 20th examination was 45.83 years (SD 3.76). The characteristics and baseline lung function parameters of the participants are summarized in Table 2. At the time of their first medical examination, 30.4% of the storage workers were non-smokers, 34.8% had smoked in the past and 34.8% were current smokers. Participants with more than 16 h exposure did not differ from those with less hours of monthly exposure. Similarly, baseline characteristics of participants with more than 7 examinations did not differ from those with 7 or fewer examinations, except for smoking status (Table 2). None of the workers reported respiratory symptoms associated with their work in the extreme cold stores.

**Table 1:** Number of participants according to monthly exposure and number of examination and measure points

Examination no	No. of participants Monthly exposure to extreme cold			Measure points	
	≤ 16 h	> 16 h	Total	Cumulative	%
1	19	27	46	46	11.6
2	19	27	46	92	11.6
3	18	26	44	136	11.1
4	16	24	40	176	10.1
5	18	24	42	218	10.6
6	16	21	37	255	9.3
7	11	18	29	284	7.3
8	0	11	11	295	2.8
9	0	10	10	305	2.5
10	0	10	10	315	2.5
11	0	10	10	325	2.5
12	0	10	10	335	2.5
13	0	9	9	344	2.3
14	0	9	9	353	2.3
15	0	9	9	362	2.3
16	0	9	9	371	2.3
17	0	8	8	379	2.0
18	0	7	7	386	1.8
19	0	6	6	392	1.5
20	0	6	6	398	1.5

**Table 2:** Characteristics of participants at baseline

	All	Monthly exposure to cold			
	*n* = 46	≤ 16 h *n* = 19	> 16 h *n* = 27	= 7 examinations *n* = 18	> 7 examinations *n* = 11
Age in yrs. (mean, SD)	35.09 (9.34)	35.58 (9.88)	34.74 (9.11)	36.33 (7.18)	35.00 (9.32)
Sex male (%)	100	100	100	100	100
Height in cm (mean, SD)	180.39 (6.67)	180.21 (7.25)	180.52 (6.36)	181.06 (7.73)	180.82 (7.21)
Weight in kg (mean, SD)	87.05 (16.91)	91.58 (17.13)	83.87 (16.32)	94.08 (17.79)	80.55 (14.48)
Hours of monthly exposure (mean, SD)	27.85 (24.67)	6.74 (5.47)	42.70 (21.86)	20.72 (17.36)	49.09 (23.68)
Smoking status					
Never smoker (%)	30.4	21.1	37.0	5.6	45.5
Former smoker (%)	34.8	42.1	29.6	50.0	36.4
Current smoker (%)	34.8	36.8	33.3	44.4	18.2
FEV_1_ in %-pred. (mean, SD)	94.72 (12.93)	93.91 (13.48)	95.29 (12.76)	93.46 (14.86)	98.37 (13.32)
FVC in %-pred. (mean, SD)	96.63 (11.82)	94.89 (10.83)	97.86 (12.52)	95.84 (13.40)	99.44 (13.10)
FEV_1_/FVC in %-pred. (mean, SD)	97.46 (5.89)	98.21 (4.66)	96.93 (6.66)	96.86 (5.16)	98.42 (4.63)
D_L,CO_ in %-pred. (mean, SD)	94.42 (13.34)	93.06 (12.34)	95.65 (14.40)	90.28 (13.39)	97.05 (9.19)
D_L,CO_/VA in %-pred. (mean, SD)	94.41 (13.17)	91.74 (11.61)	96.82 (14.29)	89.36 (11.32)	96.99 (15.58)

D_L,CO_: diffusion capacity for CO, D_L,CO_/VA: Krogh-factor, FEV_1_: forced expiratory volume in the first second, FEV_1_/FVC: Tiffeneau-Index, FVC: forced vital capacity

The mean values of the outcome parameters at baseline (1st check-up) and by the 10th and 20th examinations are presented in Table 3. The comparison over time shows an increase of the mean %-predicted values of FEV_1_, FVC and FEV_1_/FVC and a decline in the %-predicted of the diffusion capacity (D_L,CO_) with increasing number of medical examinations.

**Table 3:** Mean values (%-predicted) at 3 time points

	1st examination (*n* = 46)		10th examination (*n* = 10)		20th examination (*n* = 6)	
	Mean	SD	Mean	SD	Mean	SD
FEV_1_ (%-pred.)	94.72	12.93	100.54	13.16	99.55	8.49
FVC (%-pred.)	96.63	11.82	105.79	7.43	101.25	6.34
FEV_1_/FVC (%-pred.)	97.46	5.89	94.37	8.44	97.91	4.21
D_L,CO_ (%-pred.)	94.42	13.35	95.10	27.21	86.87	9.43
D_L,CO_/VA (%-pred.)	94.41	13.17	102.27	17.50	98.21	10.90

D_L,CO_: diffusion capacity for CO, D_L,CO_/VA: Krogh-factor, FEV_1_: forced expiratory volume in the first second, FEV_1_/FVC: Tiffeneau-Index, FVC: forced vital capacity

**Fig. 1:**
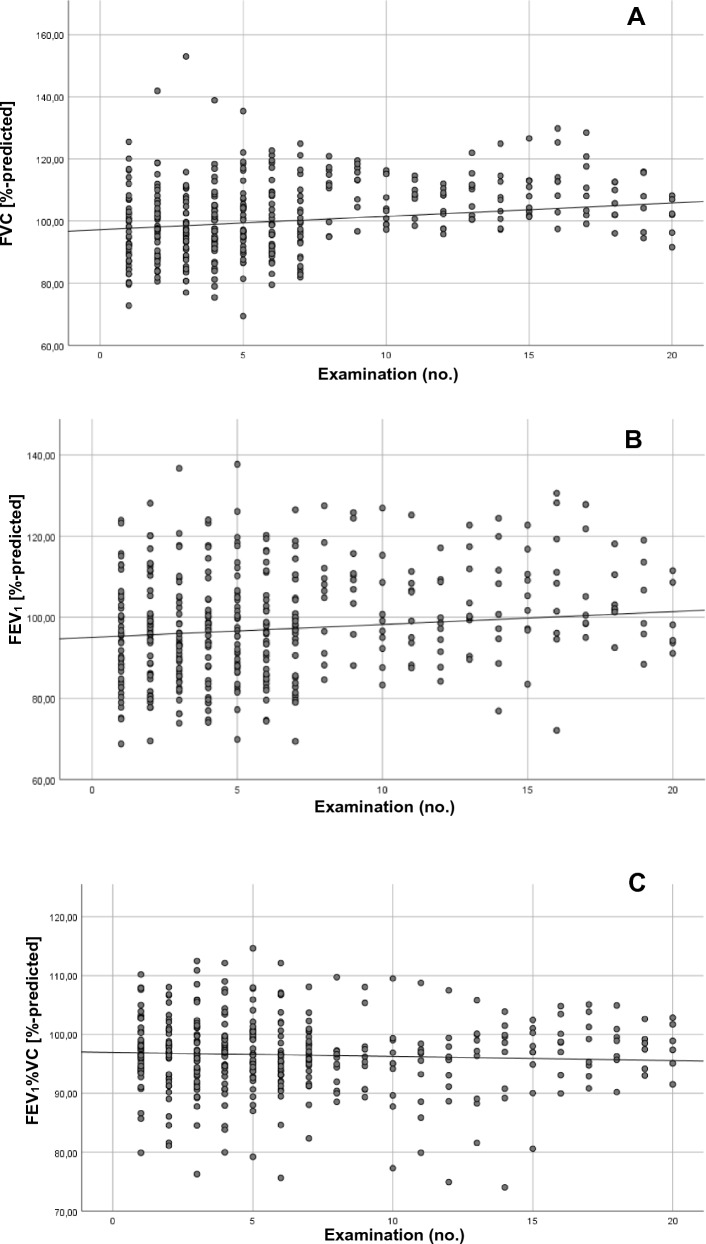
Scatter plot of spirometry parameters by measurement cluster. **a** Forced vital capacity (FVC) as %-predicted. **b** Forced expiratory volume in 1 second (FEV_1_) as %-predicted. **c** Tiffeneau-index (FEV_1_/FVC) as %-predicted

**Fig. 2:**
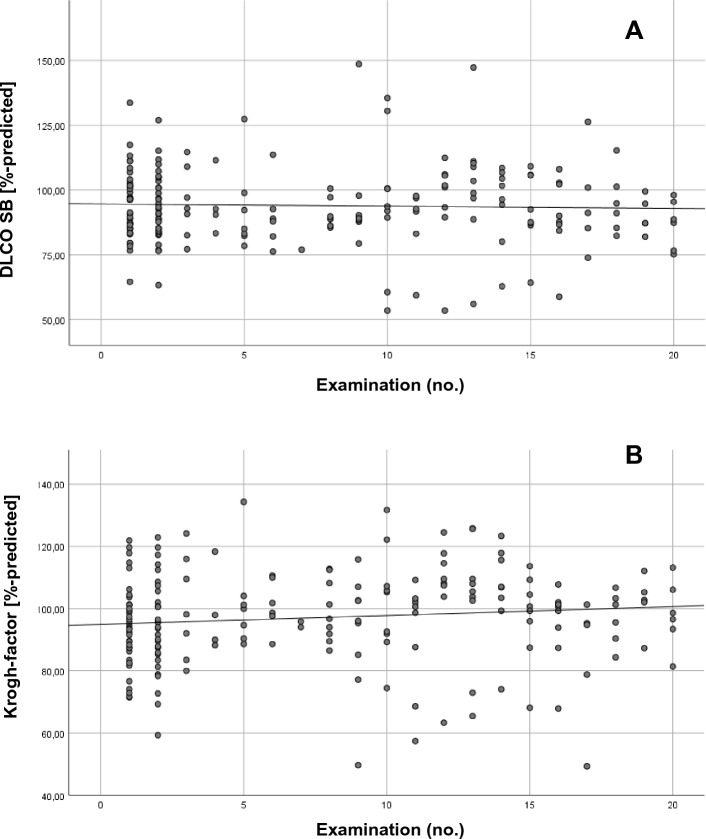
Scatter plot of diffusion by measurement cluster. **a** D_L,CO_ as %-predicted. **b** D_L,CO_/VA as %-predicted

Figure 1 shows the results of the spirometry parameters in each measurement time point. Each plot represents the results of the measurement of one worker. With growing numbers of examinations there is a trend towards increasing mean FVC %-predicted and mean FEV_1_%-predicted in each examination (see trend lines in Fig. 1a and 1b). Such a trend was not observed for the Tiffeneau-Index in % of predicted value (see Fig. 1c). Figure 2 shows the scatter plots for the parameters of the gas exchange. While mean D_L,CO_ %-predicted remains constant over time (Fig. 2a), mean D_L,CO_/VA %-predicted shows a slight increasing trend (see Fig. 2b).

The number of hours of monthly exposure to extremely cold temperatures was not associated with lung function measures (Table 4). At baseline (i.e. 1st examination) diffusion parameters as well as FVC %-predicted were higher among those exposed to cold more than 16 h per week, while FEV_1_%-predicted and FEV_1_/FVC %-predicted were lower, but the differences were not statistically significant (Table 4). Current smokers had lower values for spirometry and diffusion capacity parameters at baseline than former smokers, who in turn had lower values than never smokers, however the differences were not statistically significant except for D_L,CO_/VA %-predicted (Table 5).

**Table 4:** Lung function estimates in the linear mixed model analysis according to monthly exposure time to extreme cold temperatures

Parameter	Intercept at 1st examination (95% CI)		Difference (*p*-value)	Slope (95% CI)
	≤ 16 h	> 16 h		
FEV_1_ (%-pred.)	95.52 (90.30–100.73)	94.70 (90.28–99.13)	0.82 (*p* = 0.809)	0.32 (0.15 to 0.48)
FVC (%-pred.)	95.68 (90.99–100,37)	98.36 (94.39–102.34)	− 2.67 (*p* = 0.379)	0.43 (0.28 to 0.57)
FEV_1_/FVC (%-pred.)	98.73 (96.14–101.32)	95.69 (93.50–97.88)	3.04 (*p* = 0.075)	− 0.07 (− 0.13 to 0.00)
D_L,CO_ (%-pred.)	93.85 (87.55–100.15)	95.19 (89.52–100.87)	− 1.34 (*p* = 0.748)	− 0.10 (− 0.42 to 0.22)
D_L,CO_ VA (%-pred.)	93.93 (87.96–99.90)	95.66 (90.31–101.01)	− 1.73 (*p* = 0.661)	0.27 (− 0.07 to 0.61)

CI: confidence interval, D_L,CO_: diffusion capacity for CO, D_L,CO_/VA: Krogh-factor, FEV_1_: forced expiratory volume in the first second, FEV_1_/FVC: Tiffeneau-Index, FVC: forced vital capacity, SD: standard deviation

**Table 5:** Lung function estimates in the linear mixed model according to smoking status

Parameter	Intercept at 1st examination (95% CI)			Difference (*p*-value)		Slope (95% CI)
	Never smoker (N)	Former smoker (F)	Current smoker (C)	N–C	F–C	
FEV_1_ (%-pred.)	99.56 (93.69–105.42)	94.12 (88.67–99.58)	92.08 (86.62–97.53)	7.88 (p = 0.053)	1.95 (p = 0.610)	0.32 (0.15 – 0.48)
FVC (%-pred.)	101.11 (95.76–106.46)	96.07 (91.09–101.04)	95.10 (90.12–100.08)	5.828 (p = 0.111)	1.10 (p = 0.752)	0.43 (0.28 to 0.58)
FEV_1_/FVC (%-pred.)	97.46 (94.35–100.56)	97.30 (94.41–100.20)	96.17 (93.27–99.10)	1.834 (p = 0.373)	0.95 (p = 0.629)	− 0.07 (− 0.14 to − 0.00)
D_L,CO_ (%-pred.)	97.08 (91.10–103.05)	96.34 (89.82–102.85)	90.38 (81.65–99.11)	6.69 (p = 0,183)	5.57 (p = 0.250)	-0.11 (-0.42 – 0.19)
D_L,CO_ VA (%-pred.)	100.24 (93.98–106.49)	96.97 (91.05–102.89)	87.82 (81.64–94.00)	12.26 (p = 0.006)	8.61 (p = 0.043)	0.23 (-0.10 – 0.56)

C: current smoker, CI: confidence interval, D_L,CO_: diffusion capacity for CO, D_L,CO_/VA: Krogh-factor, F: former smoker, FEV_1_: forced expiratory volume in the first second, FEV_1_/FVC: Tiffeneau-Index, FVC: forced vital capacity, N: never smoker, SD: standard deviation

Analysis of the linear mixed model showed that all lung function parameters had values above the lower limit of normality at the first examination, with FVC showing the highest (97.25%-predicted 95% CI 94.15 to 100.34) and D_L,CO_ showing the lowest value (94.59%-predicted 95% CI 90.32 to 98.87) (Table 5). In the multivariate model including smoking status and monthly intensity of cold exposure (≤ 16 h/month vs. > 16 h/month) FEV_1_%-predicted and FVC %-predicted had a statistically significant positive slope (FEV_1_, 0.32% 95% CI 0.16% to 0.49% *p* < 0.001; FVC 0.43% 95% CI 0.28% to 0.58% *p* < 0.001), confirming the trend observed in the bivariate analysis. Including exposure time as a continuous variable in the model yielded similar results (Table 6). The positive slope of the %-predicted indicates that the absolute value of FEV_1_ and FVC decreased less over time than expected according to age of the workers. The other lung function parameters (FEV_1_/FVC %-predicted, D_L,CO_ %-predicted, D_L,CO_/VA %-predicted) showed no statistically significant change over time (Table 6).

**Table 6:** Estimates of lung function in the linear mixed model considering the influence of smoking and monthly exposure time to extreme cold temperatures

Parameter	Intercept at 1st examination (95% CI)	Slope (95% CI)	*p*	Adjusted slope (95% CI)	*p*	Adjusted slope* (95% CI)	p	Restricted to first 7 examinations	*p*
FEV_1_ (%-pred.)	95.04 (91.63 – 98.46)	0.32 (0.15 to 0.48)	< 0.001	0.32 (0.16 to 0.49)	< 0.001	0.32 (0.15 to 0.49)	< 0.001	0.25 (− 0.18 to 0.68)	0.255
FVC (%-pred.)	97.25 (94.15–100.34)	0.43 (0.28 to 0.58)	< 0.001	0.43 (0.28 to 0.58)	< 0.001	0.41 (0.20 to 0.62)	< 0.001	0.29 (− 0.30 to 0.88)	0.333
FEV_1_/FVC (%-pred.)	96.95 (95.21–98.69)	− 0.07 (− 0.14 to − 0.00)	0.049	− 0.07 (− 0.13 to 0.00)	0.057	− 0.06 (− 0.15 to 0.03)	0.193	− 0.08 (− 0.32 to 0.17)	0.540
D_L,CO_ (%-pred.)	94.59 (90.32–98.87)	− 0.09 (− 0.40 to 0.22)	0,579	− 0.11 (− 0.42 to 0.21)	0.513	− 0.14 (− 0.56 to 0.28)	0.502	− 0.47 (− 2.12 to 1.19)	0.573
D_L,CO_/VA (%-pred.)	94.89 (90.83–98.96)	0.29 (− 0.05 to 0.62)	0.090	0.22 (− 0.11 to 0.56)	0.201	0.39 (0.02 to 0.76)	0.038	− 0.68 (− 2.11 to 0.75)	0.347

*CI* confidence interval, *D*_*L,CO*_ diffusion capacity for CO, *D*_*L,CO*_*/VA* Krogh-factor, *FEV*_*1*_ forced expiratory volume in the first second, *FEV*_*1*_*/FVC* Tiffeneau-Index, *FVC* forced vital capacity, *SD* standard deviation

All models adjusted for smoking status and monthly exposure (≤ 16 h, > 16 h)

*Adjsuted for smoking status and monthly exposure [h]

Restricting the analysis to the first 7 examinations resulted in a loss of statistical significance for the slope of all parameters, while the trend remained the same except for D_L,CO_/VA %-predicted.

## Discussion

### Main results

After a median follow-up of 3.5 yrs. (max. 10 years) and accounting for age, smoking status and monthly exposure time to extremely cold temperatures, we found no deterioration beyond aging in any of the lung function parameters studied (%-predicted of FEV_1_, FVC, FEV1/FVC, D_L,CO_ or D_L,CO_/VA, resp.). Thus, there was no evidence for the development of obstructive or restrictive ventilation disorders associated with the occupational intermittent exposure to extremely cold indoor temperatures (− 55 °C) compared with the general population. As it could be expected, smokers had lower values for spirometry and diffusion capacity parameters, although in our sample the difference was statistically significant only for D_L,CO_/VA %-predicted.

### Interpretation

The association between occupational exposure to cold temperatures and lung function has been little studied. Shiryaeva et al. (2015) found FEV_1_, FVC and FEV_1_/FVC within the limits of normality among trawler fishermen and workers in salmon processing plant who were regularly exposed to moderately cold temperatures (Shiryaeva et al. 2015). Although the findings of this study, like ours, suggest no association between cold temperatures and lung function, their cross-sectional design does not allow to draw conclusions about the development of lung function parameters over time. In a 12-month follow-up, Jammes et al. (2002) observed a slight decrease in FEV_1_ and an increase in airway resistance among cold storage workers exposed to temperatures between + 3 °C and + 10 °C daily for almost the entire working time, with 25% of their working time at + 3 °C (Jammes et al. 2002). The mean FEV_1_ of exposed workers dropped form 114% of the predicted value to 95% of the predicted value (Jammes et al. 2002). Our results cannot confirm these findings. This discrepancy may be explained by differences in the length of exposure due to the type of work. The workers studied by Jammes et al. spent continuously 6 h daily in the cold rooms (+ 10 °C) and intermittently 30 to 60 min in the colder refrigerators (+ 3 °C) while the storekeepers in our study—although working in significantly colder temperatures (− 55 °C)—were exposed only intermittently and for a maximum of 30 min. Irritative and inflammatory effects of cold on the lower respiratory tract have been observed in individuals exposed to cold for several hours daily without interruption, such as endurance winter athletes (Sue-Chu 2012) or outdoor workers (Kontaniemi et al. 2003; Stjernbrandt et al. 2022). The effects of cold on the respiratory tract also appear to be related to higher oxygen uptake and tidal volumes (Sue-Chu 2012), which arise during physically demanding work as in most outdoor occupations or endurance training. Cold storekeeping has been shown to be a physically demanding activity, with oxygen uptake close to the endurance limit when workers are moving loads of up to 15 kg in the frozen food industry (Groos et al. 2021). The workers in our sample, however, worked with smaller loads (packages of 1–2 kg weight), thus we would not expect high oxygen uptake during the time spent in ultra-cold storage.

It has been shown, that the cooling of facial skin triggers bronchoconstriction during exposure to cold temperatures (Koskela 2007). The workers in our sample weared protective clothing which cover large parts of the face (i.e. winter hat with earflaps, neck gaiters), although we do not have systematic information about the use of these clothing. The correct use protective facial clothing may have reduced facial cooling reflexes in our sample.

### Limitations

The main limitation of our study concerns the number of participants. Although we had an acceptable number of subjects (*n* = 46) who had worked in the ultra-cold warehouse for least one year, only six of them worked continuously in the extremely cold storage over the full 10 years of follow-up due to staff fluctuation. Like in other studies on occupational health, we cannot rule out a healthy-worker effect, which would arise when unhealthier workers leave over time and healthier are retained (Chowdhury et al. 2017). Our results suggest that the natural decline of FEV_1_ and FVC in our sample was less accentuated than expected according to age: we observed better values for the parameters FEV_1_%-predicted and FVC %-predicted at the end of follow-up, i.e. compared to the reference values of the same age group. Particularly, the age-adjusted loss of mean FEV_1_ and FVC were less in those who worked for 10 years than in the total collective (see Table 3). This suggests, that those with better lung function worked longer. At baseline, workers with longer follow-up had higher values in %-predicted for all lung function parameters than workers with only 7 examinations, although the differences were not statistically significant (Table 2). Regarding smoking status, there were more never smokers among workers with longer follow-up. In addition, those who were smokers among the participants working for 10 years quitted smoking to some timepoint of the study, as indicated by the proportion of active smokers at the moment of the 20th check-up (0%) in comparison to the proportion of active smokers at the moment of the 1st check-up (35%). Thus, it is conceivable that healthier subjects have contributed more data to the study than less healthy. However, we do not have any indication, that subjects leaving the job during the 10-year follow-up period did so because of respiratory disease – although this information was not recorded systematically. The main loss of subjects to the study (*n* = 11) was due to the cessation of the special extended health surveillance at our institute for those workers with less than 16 h per month of exposition to extreme cold. The researchers did not have any influence on this decision. Baseline characteristics and %-predicted spirometry and diffusion parameters of the workers with less than 16 h monthly exposure did not statistically significantly differ from the values of those exposed more than 16 h monthly (see Tables 2 and 4). In addition, we performed a sensitivity analysis restricting the sample to the first 7 examinations. As in the analysis with the whole sample, we found a trend to higher values in %-predicted for FEV_1_ and FVC and a trend to lower %-predicted FEV_1_/FVC and D_L,CO_, although none was statistically significant. The only parameter that showed an inverse trend was D_L,CO_/VA, also not statistically significant (see Table 6). Thus, we do not think that the loss to follow-up influenced our results relevantly, and in particular, we do not expect an overestimation of the values in %-predicted due to the loss of follow-up.

### Strengths

To our knowledge, this is the first study addressing the potential long-term effects of occupational exposure to extreme cold temperatures on spirometry parameters and diffusion capacity over a longer period of time. Previous studies on occupational cold exposure have focused on the association with respiratory symptoms, but not on lung function data (Piedrahita et al. 2008; Ghani 2020; Stjernbrandt et al. 2021; Stjernbrandt et al 2022). Other studies, which monitored lung function parameters and occupational cold exposure, were either cross-sectional (Shiryaeva et al. 2015) or had a short follow-up (Jammes et al. 2002). However, in the present study, the semiannual surveys over 10 years allowed a substantial number of measurement points (*n* = 398) to be included in the multivariate analysis. Another strength of the study is that all lung function measurements were performed at the same institution, following the same standards over time.

Smoking status and age were considered at each visit and could be included in the multivariate analysis. The presentation of the results as %-predicted allowed to evaluate the lung function changes over time, regardless of the age of the subjects.

Finally, we measured lung function parameters after an exposure-free period of at least 12 h. Thus, our measurements are not influenced by potential immediate effects of cold in the airways.

## Conclusions

Long term intermittent occupational exposure to extreme cold temperatures (− 55 °C) does not appear to cause irreversible deleterious changes in lung function in healthy workers, thus the development of obstructive or restrictive lung diseases is not expected.

Limiting the duration of stay in the deep cold storage to a maximum of 30 min each time entering the exposure area seems to protect workers from long-term pulmonary damage, at least from those that can be detected by spirometry and diffusion capacity measurements. Since we cannot rule out a relevant healthy-worker effect, further research is needed to confirm our findings.

Nevertheless, according to our results it seems acceptable to expand the interval of the health surveillance examination with lung-function tests of these workers to 12 months instead of the current interval of 6 months which had been previously established for precautionary reasons.
